# Multi-Criteria Decision Making Methods for Selection of Lightweight Material for Railway Vehicles

**DOI:** 10.3390/ma16010368

**Published:** 2022-12-30

**Authors:** Varun Sharma, Fatima Zivic, Dragan Adamovic, Petar Ljusic, Nikola Kotorcevic, Vukasin Slavkovic, Nenad Grujovic

**Affiliations:** 1 Faculty of Engineering, University of Kragujevac, 34000 Kragujevac, Serbia; 2 AMM Manufacturing, 34325 Kragujevac, Serbia

**Keywords:** multicriteria decision-making methods, railway vehicles, dual-phase 600 steel, transformation-induced plasticity (TRIP) 700 steel, twinning-induced plasticity (TWIP) steel, aluminium alloy, Al 6005-T6, aluminium alloy, Al 6082-T6, porous aluminium (with closed cell porosity)

## Abstract

This paper deals with the selection of the optimal material for railway wagons, from among three different steel and three aluminium based materials, by using four different Multicriteria Decision Making Methods (MCDM) and comparing their ranking of the materials. We analysed: Dual-Phase 600 steel, Transformation-Induced Plasticity (TRIP) 700 steel, Twinning-Induced Plasticity (TWIP) steel, Aluminium (Al) alloys, Al 6005-T6, and Al 6082-T6, and porous Al structure with closed cells. Four different MCDM methods were used: VIKOR, TOPSIS, PROMETTHEE and the Weighted aggregated sum product assessment method (WASPAS). Key material properties that were used in the MCDM analysis were: density, yield strength (Y.S.), tensile strength (T.S.), Y.S./T.S. ratio, Youngs modulus (Y.M.), cost and corrosion resistance (C.R.). Research results indicate that aluminium and its alloys prove to be the most suitable material, based on setup criteria. Advanced steels also achieved good ranking, making them a valid option, immediately behind lightweight aluminium alloys. Porous aluminium did not perform well, according to the used MDCM methods, mainly due to the significantly lower strength exhibited by the porous structures in general.

## 1. Introduction

Selection of materials for railway vehicles represents a complex task, due to many suitable material candidates, among which both aluminium alloys [1,2,3,4] and advanced high strength steels (AHSSs) [5,6] offer different benefits for vehicle body elements and structure and are both applied with the focus on lightweight structures. 

AHSSs have been used in structural elements within the railway and automotive industries for a long time, due to their superb properties in relation to combined mechanical strength and ductility. Research and improvement of the 3rd generation of AHHSs is aiming towards a novel 4th generation of AHHSs that can have specifically tailored material properties and provide a lightweight design [5]. Different approaches—for increasing the mechanical strength while reducing the weight and cost of AHSSs for transportation industries—have been reviewed [6,7]. Development of dual phase (DP) steels, transformation induced plasticity (TRIP) steels, and twinning induced plasticity (TWIP) steels, among other types of AHSSs—are of significant importance for automotive applications [8,9] for improving crashworthiness and formability with lightweight properties. Processing and tailoring of TRIP steel properties to achieve a desired design have shown that it is a promising candidate for structural components [10]. Microstructure of the TWIP steels provides improved ductility at high strength [11]. One of the challenges in the development of AHSSs is hydrogen embrittlement (HE) since it can induce irreversible damage and catastrophic failures in the material structure over time [12,13], and different methods have been used to detect and overcome this issue [14]. Moreover, profound understanding of the corrosion mechanisms in these steels is important for their further development [11]. In general, welding is one of the major technologies utilized in elements of railway, automotive, and aerospace industries and different aspects related to the welding of AHSSs are studied [15,16] to provide important data on their functional behaviour aiming at improvements in AHSSs. Material cracking within the weld and heat affected zone is still an issue with AHHSs, due to a difference of fatigue and tensile strength between the AHSSs and the joint [16].

Different series of aluminium alloys have already been heavily used in elements of railway vehicles, and 6005 and 6082 aluminium alloys are amongst the most often used ones in the 6xxx alloys series [1,2,17,18], due to their superb lightweight properties [19] and corrosion resistance [20]. Processing routes and treatments have the most significant influence on the microstructural properties and mechanical properties of aluminium alloys [17,19,21,22]. Moreover, welding introduces changes in microstructural properties of these alloys: this is why the study of friction stir welding is currently attracting particular interest [18,23]. The 6005 and 6082 aluminium alloys show good mechanical properties under cyclic loading [1,24]. For elements subjected to the loading due to vibrations and shock, 6082 aluminium alloys have been recommended [19].

New developments in material research have introduced porous material structures including porous aluminium structures [3,25,26], in order to further lower the weight of the components. Aluminium foams with open-cell structure have shown exceptional properties in the context of applications that involve energy and sound absorbing, electromagnetic shielding or controlled heat exchange [3], and in railway, automotive and aerospace industries. Their mechanical properties can be tailored, by adjusting the level of porosity, cell shapes and distribution patterns, and the thickness of the struts and walls. Porous aluminium foams are especially suited for shock absorbing elements [27]. Further improvement of the mechanical strength and increase in stiffness can be achieved by incorporating different fillers or composite structures. Accordingly, porous aluminium (Al) composites for lightweight structural applications in transportation-related industries, including railway applications, have gained attention in recent years [4,28]. Al foam has already been commercially used to support bumper elements or wagon crumple zones (to absorb energy during possible collision) in some railway vehicles [4,29]. Some new approaches in the production of Al foams have been studied, such as a friction stir processing route [30]. Sandwich panels that incorporate aluminium foams are also recognised as very important material structures for different functional elements in automotive and railway components [31]. However, metal foams in general need further investigation from different perspectives, and especially their cost for mass production still prevent them from wider functional applications [4]. Welding of components made of porous structures, including novel friction stir welding, is also of significant importance for real applications [32].

It is rather hard for the designers of new or updated vehicles to select the appropriate material from the comprehensive list of available materials, especially considering that each of the previously mentioned material classes offer some benefits, yet at the same time all have shown certain drawbacks. Significant parameters and influential factors can vary widely depending on the specific component; and its function and parameter optimization can require a number of methods and approaches, in which multicriteria decision-making in material selection is now a necessity [33,34,35,36]. It is important to analyse the potential cost of the structure and the effect that uncertainties related to material strength could have on it, especially in industrial decision-making. Using available information regarding material properties, it is possible to form a data-driven model which creates a correlation between input parameters and system objectives [33]. There is a wide range of materials all with their own properties, but inadequate choice often leads to larger costs and ultimately can result in product failure [37,38]. Since many different factors should be considered, multicriteria decision-making (MCDM) methods are used to predict the impact that they can have, thus narrowing down the best possible solution out of many available ones [34]. Alternative techniques such as Ashby’s graphical techniques or digital tools including GRANTA CES selector and MATWEB have also been used in material selection [39]. The MCDM method evaluates strengths and weaknesses of all considered materials, compares them and ranks them based on their economic, technical and environmental results [36]. Aside from material properties, MCDM methods can be used for evaluation of the optimal solution for the manufacturing processes, also ranking them based on their performance in terms of desired attributes [40]. Many different methods have been developed, such as: TOPSIS (Technique for Order Performance by Similarity to Ideal Solution) [41], Weighted aggregated sum model [37], PROMETHEE (Preference ranking organization method for enrichment evaluation) [42] and VIKOR [43,44].

Their implementation has been involved in different areas of engineering such as energy, material, operation research, and safety management [45]. VIKOR is a multicriteria ranking method and calculates the best (compromise) solution in a multicriteria environment from the set of X feasible alternatives (Y1, Y2,…,YX) evaluated based on the set of n criterion functions [46]. The VIKOR method has been widely used in optimisation of concrete structures and for industrial robot selection [47,48]. Further development showed that the regret theory-based compromise ranking method showed better performance compared to the original compromise ranking method [48]. PROMETHEE has been applied for the identification of the best material out of a large number of alternatives having conflicting criteria [42]. It is based on a multicriteria net flow which includes preferences and indifferences. Moreover, a fuzzy PROMETHEE approach was proved to be an efficient and feasible tool for material selection [49]. Hybrid MCDM methods that can combine DEMATEL (Decision Making and Evaluation Laboratory), GRA (Grey Relational Analysis), ANP (Analytical Network Process) and TOPSIS (Technique for Order Performance by Similarity to Ideal Solution) also proved to be useful for material optimisation [39].

This paper is focused on selection of the optimal material for a railway wagon, from three different steel and three aluminium based materials, using four different MCDM methods and comparing their ranking of the materials. We set up ranking criteria for: Dual-Phase DP 600 steel, Transformation Induced Plasticity TRIP 700 steel, Twinning Induced Plasticity, TWIP steel, Aluminum, Al 6005-T6, Aluminum, Al 6082-T6, and porous Al structure with closed cells. Four different Multicriteria decision-making methods (MCDM) were used: VIKOR, TOPSIS, PROMETTHEE and Weighted aggregated sum product assessment method. Key material properties that were used in the MCDM analysis were: density, yield strength (Y.S.), tensile strength (T.S.), Y.S./T.S. Ratio, Young’s Modulus (Y.M.), cost and corrosion resistance (C.R.).

## 2. Materials and Methods

### 2.1. Material

**Figure 1:**
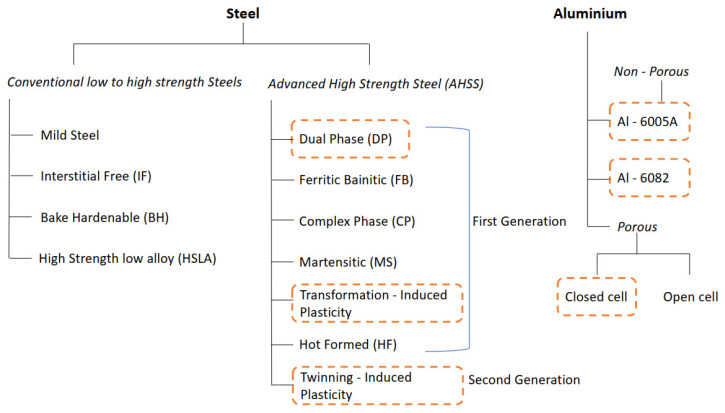
Prospective engineering material for rail wagon light vehicle.

Current research aims to continue to expand the broad spectrum of advanced high strength steel (AHSS) in light wagon railway engineering applications. Their incorporation has grown exponentially due to their safety, fuel efficiency, and the ease of manufacturability. First and second-generation AHSS are being applied widely on their own. Detailed categorized presentation of conventional low to high strength steel and advanced high strength steel is presented in Figure 1. Formation of dual-phase materials (DP) is now also carried out from low or medium carbon steel under thermomechanical processing. Their development began in the 1970s when the metallurgical industry was searching for steel with high strength and ductility [50,51]. Their microstructure comprises a ferrite phase (which induces low initial yield stress) and a martensite phase (causing high ultimate tensile strength). The microscopic behaviour of DP can be controlled by various parameters such as volume fractions, morphology, size, aspect ratio, interconnectivity, etc. [51]. These impose strain hardening and homogenous plastic flow [52]. 

Further improvement in AHSS has led to the foundation of transformation-induced plasticity steel (TRIP). This is known for its dual optimal properties, i.e., strength and ductility. Its microstructure comprises austenite with sufficient thermodynamic instability; and it contains high values of carbon, silicon, and aluminium. TRIP exhibits large uniform elongations, high fracture toughness, and strength. Its property is strongly influenced by the deformation-induced martensitic transformation from the parent phase (FCC γ austenite) to the product phase (BCC α’ martensite), which depends upon the applied stress, deformation history, strain rate, temperature, composition, and other factors [53,54]. Subsequent enhancement of AHSS directed the progress of twinning-induced plasticity (TWIP) steels which are also known as second-generation steel. They fall under the category of austenite steel whose deformation phenomena depend on mechanical twining as well as the glide of individual dislocations [55]. They are widely known for their outstanding mechanical properties such as high tensile strength and ductility. Moreover, they contain a large concentration of manganese. The quantitative study of their deformation twinning strongly depends upon the nucleation and growth process. They have better resistance to corrosion and wear, high energy absorption, and crash safety [56]. As discussed previously, AHSS is compared with other grades of aluminium, i.e., Aluminium 6005 and 6082. Both belong to the wrought aluminium-magnesium silicon family. They are mostly formed through an extrusion and rolling process. Their development is precisely done by heat treatment to produce tempers with higher strength [57]. In addition to AHSS and aluminium, a class of structural-functional materials—“Porous structures”—is also added to the list for material evaluation. They are an optimal index of mechanical and physical properties, due to their porous nature. They can be seen in the field of energy management, heat insulation, fluid filtration, vibration suppression, etc. Highly porous materials have relatively low mass density, high structural rigidity, large exchange surface (102–104 m^2^/m^3^), good resistance to thermal shocks, high pressures, high temperatures and thermal cycling, excellent absorption of mechanical shock and electromagnetic dumping—hence why these structures themselves suit well to structural bodies [58,59]. They are advanced and supreme, though present certain challenges. They contain a number of voids or pores which are interconnected to each other through a bone-shaped rod called a ligament while their skeletal structure is called the matrix. In order to make these structures, a highly dense matrix is arranged in a schematic of rows and columns. The characteristic of the porous materials varies depending on its composition, shape, pore arrangement and also on the size of the pores. A porous material must have two essential properties: first, the material must contain a lot of pores; and secondly, the pores should be designed specifically in order to achieve the expectancy index of the material’s performance. The engineering property of a porous structure in closed cell format is illustrated in Table 1, as well as for the other materials observed in our study. Chemical composition of DP 600, TRIP 700 and TWIP steel are shown in Table 2. The chemical composition of 6005-T6 and 6082-T6 aluminium is presented in Table 3.

### 2.2. Methods

#### 2.2.1. VIKOR Method

The algorithm of the VIKOR method encompasses the following steps:

Step 1: Finding the best $f_{i}^{*}$ and the worst $f_{i}^{–}$ values of all norms, i.e., deck condition rating, superstructure condition rating and other quantitative criteria, *i* = 1,2,…,*n*. If the *i*th norms represent a benefit (the larger, the better), then $f_{i}^{*}$ = $max_{j}f_{ij}$ and $f_{i}^{-}$ = $min_{j}f_{ij}$; if the *i*th norms represent a cost (the smaller, the better) then

(1) $$f_{i}^{*}=min_{j}f_{ij}\;\mathrm{and}\;f_{i}^{-}=max_{j}f_{ij}$$

Step 2: Determining the value $S_{j}$ and $R_{j}$ by the relations:

(2) $$S_{j}=\sum_{i=1}^{n}w_{i}\left(f_{i}^{*}-f_{ij}\right)/\left(f_{i}^{*}-f_{i}^{-}\right)$$

(3) $$R_{j}=max_{i}\;[w_{i}\left(f_{i}^{*}-f_{ij}\right)/\left(f_{i}^{*}-f_{i}^{-}\right)$$

where $S_{j}$ and $R_{j}$ represent the utility and regret measure, respectively, and $w_{i}$ is the weight of the ith criterion. The obtained solution for $min_{j}R_{j}$ is the smallest distinct regret of the opponent [1].

Step 3: Calculating the VIKOR index $Q_{j}$:

(4) $$Q_{j}=\frac{v\;\left(S_{j}-S^{*}\right)}{\left(S^{-}-S^{*}\right)}+(1-v)\frac{\left(R_{j}-R^{*}\right)}{\left(R^{-}-R^{*}\right)}$$

where $S^{*}$= $min_{j}S_{j}$, $S^{-}=max_{j}S_{j},\;\;R^{*}=min_{j}R_{j},\;R^{-}=max_{j}R_{j},\;S_{j}$ and $R_{j}$ are calculated in step 2 and v is introduced as a weight strategy of the ‘majority of criteria’ (or ‘the maximum group utility’), here v = 0.5.

Step 4: Ranking the order of preference by the value Q.

The smallest value obtained from the VIKOR value is considered to be the best value. A solution near to the ideal point is proposed, based on the ranking value. 

#### 2.2.2. A Technique for Order of Preference by Similarity to Ideal Solution—TOPSIS

Step 1: Building the decision matrix as:

(5) $${\left[f_{mp}\right]}_{M\times P}$$

Step 2: Calculating the aggregation values by the average value procedure.

Step 3: Normalizing the decision matrix ${\left[r_{mp}\right]}_{M\times P}$ obtained using the linear normalization procedure:

(6) $$r_{mp}=\frac{f_{mp}}{\sum_{1}^{M}fmp}$$

Step 4: Finding the Positive Ideal Solution (PIS), $d_{mp}{}^{+}$ and Negative Ideal Solution (NIS), $d_{mp}{}^{-}$:

(7) $$\mathrm{For}\;\max:\;d_{mp}{}^{+}=\max value\;\mathrm{and}\;d_{mp}{}^{-}=\min value$$

(8) $$\mathrm{For}\;\min:\;d_{mp}{}^{+}=\min value\;\mathrm{and}\;d_{mp}{}^{-}=\max\;value$$

Step 5: Separating the measures, from Positive Ideal Solution (PIS), $d_{mp}{}^{+}$ and Negative Ideal Solution (NIS), $d_{mp}{}^{-}$:

(9) $$d_{M}{}^{+}=\overset{M}{\underset{1}{\sum }}W_{p}\cdot \left|d_{mp}{}^{+}-r_{mp}\right|$$

(10) $$d_{M}{}^{-}=\overset{M}{\underset{1}{\sum }}W_{p}\cdot \left|d_{mp}{}^{-}-r_{mp}\right|$$

Step 6: Calculating the performance score.

Step 7: Ranking determined according to the value of the performance score. A high closeness coefficient represents the ideal experimental run [2,3].

#### 2.2.3. PROMETHEE

The procedural steps of the PROMETHEE II method are listed below [4]:

Step 1: Constructing the decision matrix

Step 2: Calculating the normalization decision matrix using beneficial and non-beneficial equations:

(11) $$R_{ij}=\frac{\left[x_{ij}-\min(x_{ij})\right]}{\left[\max(x_{ij})-\min(x_{ij})\right]}\;\left(i\;=\;1,\;2\;\ldots ,\;m;\;j\;=\;1,\;2,\;\ldots ,\;n\right)$$

where $x_{ij}$ is the performance measure of the *i*th alternative with respect to the *j*th criterion, and $R_{ij}$ is the normalized value of $x_{ij}$. 

For non-beneficial criteria, the equation can be rewritten as follows:

(12) $$R_{ij}=\frac{\left[max\;\left(x_{ij}\right)-x_{ij}\right]}{\left[\max(x_{ij})-\min(x_{ij})\right]}$$

where $\max(x_{ij})$ and $\min(x_{ij})$ are the maximum and minimum values of $\left(x_{ij}\right)$, and *n* is the number of evaluation criteria. The purpose of performing normalization is to convert criteria values into dimensionless values. Using normalization, the criteria value lies between 0 and 1. Sometimes, partial normalization is also carried out and adopted [5]. 

Step 3: Calculating the evaluative differences of the *i*th alternative with respect to other alternatives.

Step 4: Calculating the preference functions *P_j_* (a, b) using preference thresholds and indifferences [6]. However, it is a rather challenging task to find the preference functions based on each criterion. To solve such a problem, simplified preference functions are applied here:

(13) $$P_{j}(a,\;b)=0\;\mathrm{if}\;R_{aj}\leq \;R_{bj}$$

(14) $$P_{j}(a,\;b)=(R_{aj}-R_{bj})\;\mathrm{if}\;R_{aj}\;\geq \;R_{bj}$$

Step 5: Calculating the aggregated preference function taking into account the criteria weights. The aggregated preference function, π (a, b) is

(15) $$=\left[\sum_{j=1}^{n}w_{j}P_{j}\left(a,\;b\right)\right]/\sum_{j=1}^{n}w_{j}$$

Step 6: Determining the leaving and entering outranking flows as follows:

Leaving (positive) flow for ath alternative, *φ^+^* (a)

(16) $$=\frac{1}{m-1}\sum_{b=1}^{n}\pi \;\left(a,\;b\right)\;\;\;\;(a\neq b)$$

Leaving (negative) flow for ath alternative, *φ*^−^ (a)

(17) $$=\frac{1}{m-1}\sum_{b=1}^{n}\pi \;\left(b,a\right)\;\;\;\;(a\neq b)$$

Step 7: Calculating the net outranking flow for each alternative:

*φ* (a) = *φ^+^* (a) − *φ^−^* (a) (18)

where *φ* (a) is the net outranking flow value for alternative a.

Step 8: Determining the ranking of all the considered alternatives depending on the values of *φ* (a). Thus, the best alternative is the one with the highest *φ*(a) values.

#### 2.2.4. Weighted Aggregated Sum Product Assessment Method (WASPAS)

1. Constructing the decision matrix.
2. Calculating the normalization decision matrix using beneficial and non-beneficial equations:

(19) $$Non\;beneficial=\frac{\left[\min(x_{ij})\right]}{\left[\left(x_{ij}\right)\right]}\left(i\;=\;1,\;2\ldots ,\;m;\;j\;=\;1,\;2,\;\ldots ,\;n\right)$$

where $x_{ij}$ is the performance measure of the *i*th alternative with respect to the *j*th criterion.

For beneficial criteria, the equation can be rewritten as follows:

(20) $$Beneficial=\frac{\left[\left(x_{ij}\right)\right]}{\left[\max(x_{ij})\right]}$$

This normalization procedure is required to make the criteria values dimensionless and comparable. After normalization, all the criteria values should lie between 0 and 1. In some cases, partial normalization procedure may also be adopted [61].

3. Determining the performance score according to below-mentioned equations.
  A. The weight sum method (*WSM*) is based on the weight of each criteria and the performance value as:
    (21) $$A_{i}^{WSM}=\sum_{j=1}^{n}w_{j}x_{ij}$$
    where $w_{j}$ is the weight of each criteria and $x_{ij}$ is the performance value.
  B. The weight product method (*WPM*) is based on the weight of each criteria and the performance value as
    (22) $$A_{i}^{WPM}=\prod_{j=1}^{n}{x_{ij}}^{w_{i}}$$
4. Performing addition and multiplication.
5. WASPAS ranking of the material according to: $A_{i}^{WASPAS}=0.5*A_{i}^{WSM}$ + $0.5*A_{i}^{WPM}$

#### 2.2.5. Weight Estimates in MCDM Methods

Assigning weights to different criteria while using MCDM tools is crucial. Decision makers often have difficulty obtaining the weight criteria. Likewise, weights affect the MCDM results, so assigning the proper weight is crucial for achieving quality results from MCDM tools. It is important to assign weights to each criterion when determining the choice between alternatives.

In multi criteria decision-making models, it is important to validate the decision in a systematic manner, backed by considering the varying importance of many different criteria. In the case of multi decision-making problems, a weighted decision matrix can be used for evaluating different alternatives and finding the best solution by assigning weights according to the relative importance of different characteristics. The parameters required for using a weighted decision matrix are:

1. A well-defined problem with various alternatives
2. A set of weights signifying the importance of each criterion
3. A well-defined reference against which comparisons will be made and set of alternatives to be ranked.

There are two methods for determining the weights in MCDM: (a) the objective weighting method and (b) the subjective weighting method.

In the objective weighting method, numerical methods are used to assign weights. There are various methods used in these calculations, such as the mean weight method, standard deviation, statistical variance formula, and criteria importance based on inter-criteria correlation (CRITIC). The disadvantage of the objective weighting method is that it does not take into account the subjective judgement and experience of the decision maker. In the case of the subjective weighting method, importance is given to the judgement of the decision maker, i.e., how much importance the decision maker gives to different alternative criteria in MCDM. The weights are assigned on the basis of the subjective judgement of the decision maker.

Therefore, taking into consideration all the important steps, this paper was executed using subjective judgement for assigning weights to different criteria, i.e., price, density, yield strength etc. Moreover, an additional 20% weight was given to density, Youngs modulus and corrosion resistance, and 10% weight each to the yield strength (Y.S.), price, tensile strength (T.S.) and Y.S./T.S. ratio. In addition, it was kept in mind that the sum of weights equals to 1.

- Density—20%
- Yield strength—10%
- Tensile strength—5%
- Y.S./T.S. Ratio—20%
- Youngs modulus—10%
- Price—5%
- Corrosion resistance—30%

## 3. Results and Discussions

In this paper, a light wagon railway material selection is solved using PROMETHEE, TOPSIS, VIKOR, and Weighted aggregated sum product assessment method. They are simple and easily comprehensible approaches in comparison to other popular MCDM techniques, such as Fuzzy AHP and ANP with respect to model complexity, model construction time, computational time, transparency, etc. Performance of a material strongly depends upon on its material properties. Therefore, to enhance the performance of a particular material, it is highly desirable to select the most sophisticated material with beneficial and non-beneficial values. Properties such as high tensile strength, low cost, good corrosion resistance, high yield to ultimate strength ratio, etc., are important to consider from a railway engineering point of view. In order to perform an in-depth analysis of the relative performance of the considered MCDM methods with respect to various model characteristics, different subjective judgment scales are proposed, such as for model complexity. Density and price values are considered to be lower, while the yield strength, tensile strength, Y.S./T.S., corrosion resistance, and Youngs modulus considered to be higher. Some parameters (such as corrosion resistance) were assigned one of three values for crisp measurement instead of qualitative performance values: 1—lower, 2—average, 3—good. Corresponding properties of the prospective materials for light wagon railway engineering are included in Table 4, in which P1-P2-P3-P4-P5-P6-P7 denote the parameters of density, yield strength, tensile strength, Y.S./T.S. Ratio, Young modulus, price, and corrosion resistance. The available engineering material alternatives compared are Dual Phase, DP 600, Transformation Induced Plasticity (TRIP 700), Twinning Induced Plasticity (TWIP), Aluminium (Al 6005-T6), Aluminium (Al 6082-T6), and Porous Structure (Al—Closed cell), which are denoted as M1-M2-M3-M4-M5-M6 in Table 5. Most of the values presented in Table 1 were acquired from a steel supplier. The chemical composition of different AHSSs and aluminium grades are presented in Table 2 and Table 3. 

However, selecting the right material with adequate properties remains a challenging task. Better quality and longer durability are always desired criteria, here provided by adding alloy to enhance the strength. As strength is one of the key parameters for railway application, higher-strength automatically offers higher load-bearing ability under different working conditions. Tensile strength measures the resistance of the material to break under tensions. Values of tensile strength should be as high as possible. Yield strength is the stress point at which plastic deformation is produced. Youngs modulus describes the ability of a material to withstand changes in length under tension or compressions. It is often referred to simply as elastic modulus and its value should be as high as possible. On the other hand, a railway outer body is exposed to the surrounding atmosphere, thus materials having higher corrosion resistance would be better options for this design aspect. Corrosion resistance refers to how well a substance can withstand damage caused by oxidations or other chemical reactions. Another important criterion which should be taken into consideration during the material selection process is overall price, therefore the cost of materials should be as low as possible.

### 3.1. VIKOR

VIKOR is known as a compromise ranking solution method. It is based on the agreement established by mutual concession. The first assumptions considered during the VIKOR algorithmic steps were similar to TOPSIS. They were calculated based on non-beneficial criteria (a lower value is desired) and beneficial criteria (a higher value is desired). The further steps consist of finding the best and worst value for each criterion. For beneficial criteria, the maximum value is best and minimum value is worst. For non-beneficial criteria, the minimum value is best and the maximum value is worst, as shown in Table 6. They were assigned and calculated based on Equation (1). The obtained decision matrix is presented in Table 7. Furthermore, S_I,〖R〗_I and Q_I represent the utility measure, the regret measure and VIKOR index, respectively, calculated using Equations (2)–(4) as shown in Table 8. VIKOR proposed M4 (Aluminium Al 6005-T6) as the first choice among all other available materials.

### 3.2. TOPSIS

This method is based on the concept that the best alternative should have the shortest distance (Euclidean distance from ideal solutions). The problem addressed in the paper is finding the best material for a light wagon railway out of all available alternatives, based on six different criteria: density, yield strength, tensile strength, yield strength/tensile strength ratio, Youngs modulus, price, and corrosion resistance. The decision matrix and normalized decision matrix of response can be found using equations [47,70]. The obtained normalized decision matrix value using a vector normalization procedure is shown in Table 9. The weighted normalized decision matrix with positive ideal solutions and negative ideal solutions is shown in Table 10. They were calculated based on non-beneficial criteria (a lower value is desired) and beneficial criteria (a higher value is desired) as shown in equations [42,48]. Price and density were considered to be non-beneficial, while yield strength, tensile strength, yield strength/tensile strength ratio, Youngs modulus, and corrosion resistance were employed as beneficial criteria. Furthermore, the Euclidean distance from Positive Ideal Solution (PIS), $d_{mp}{}^{+}$ and Negative Ideal Solution (NIS), $d_{mp}{}^{-}$ were calculated using equations [49,71]. The obtained values from the Euclidean distance calculation are shown in Table 11. Thereafter, a performance score was evaluated using Euclidean distance from Negative Solution divided by sum of Positive Ideal Solution (PIS), $d_{mp}{}^{+}$ and Negative Ideal Solution (NIS), $d_{mp}{}^{-}$. The final performance score with TOPSIS ranking is presented in Table 11. Higher performance scores are considered to be the best in the ranking table, whereas the lower scores signify less important materials among all the available materials. TOPSIS ranking showed that Aluminium, Al 6082-T6 (M_5_) is the material of choice, due to its low weight, affordable price and improved corrosion resistance.

### 3.3. PROMETHEE

PROMETHEE is usually designed for quantitative as well as qualitative criteria [p]. PROMETHEE II facilitates the full ranking of alternative materials in comparison to PROMETHEE I. The beginning steps consist of normalizing the evaluation matrix as shown in Table 12 using Equations (11) and (12) according to beneficial (direct) and non-beneficial (indirect) criteria. Thereafter, differences in the *i*th alternative with respect to other alternatives are presented in Table 13. The evaluation of preference functions *P_j_* (a, b) and aggregated preference functions are calculated as shown in Table 14, using Equations (13)–(15). The obtained values of aggregated preference are then presented in Table 15. Next, simple ranking can be generated based on the net outranking flow values that come from leaving and entering the outranking flows as presented in Table 16 using Equations (16)–(18). The PROMETHEE method recommended M4 as the superior material from among the other alternative materials.

### 3.4. Weighted Aggregated Sum Product Assessment Method (WASPAS)

The decision matrix was normalized and evaluated using Equations (19) and (20) based on beneficial and non-beneficial criteria as previously discussed. Obtained values are shown in Table 17 as standard quantitative normalized values. Equations (21) and (22) were applied for the calculation of weights in the normalized decision matrix. The weighted normalized decision matrix for the weight sum method (WSM) and weight product method (WPM) are presented in Table 18 and Table 19, respectively. Further evaluation was done by summation (in case of WSM) and multiplication (in case of WPM) individually in each row for the calculation of the performance score. Individual performance scores with rankings are shown in Table 20. The WASPAS analysis showed that M_4_ and M_5_ are the best candidates.

Our research study showed that the normalized decision matrix could be used to solve material selection problems for selecting the best materials for light wagon railway vehicles. Multiple MCDM techniques such as PROMETHEE, TOPSIS, VIKOR, and Weighted aggregated sum product assessment method were applied to find the best option. However, the main challenge of light wagon railway vehicles is to find the optimal blend of both primary properties (such as density, Youngs modulus and strength), and secondary properties (such as price and corrosion resistance). These properties were therefore optimized via MCDM tools. The qualitative and quantitative material selection criteria and their weight criteria were employed to find the best alternative in terms of ranking. The obtained results of PROMETHEE, TOPSIS, and Weighted aggregated sum product assessment method show that Aluminium could be a better option than steel. Aluminium served as the best materials for lighter wagons due to their corrosion resistance property, high strength, and Youngs modulus. It was found that better weight saving was obtained using aluminium alloys compared to steel [37]. Overall comparison between all methods is shown in Table 21. The research investigation also showed that the MCDM technique has the capacity to solve a complex problem, and to help researchers in taking effective choices according to the situation. These methods can be incorporated in a wide variety of engineering applications to help the decision-maker identify the preferred choice.

Material ranking in Table 21 clearly shows rather large differences in observed material candidates. If we compare resulting score values only for the group of steels, it can be seen that scores are comparable, with slightly better values for M3 (Twinning-Induced Plasticity, TWIP steel). TWIP steel is developed aiming at better plasticity of the material, in order to provide high energy absorption in automotive applications [72,73,74]. Strain-hardening twins are generated through atomic displacements when TWIP steel is under deformation [72,73]. Twin boundaries serve as grain boundaries, thus resulting in higher strength and ductility. Yield and tensile strength of the TWIP steel are both significantly higher than all other material candidates in this study (Table 2). However, the ratio of yield to tensile strength (Y.S./T.S. ratio) also has a higher value (0.75), meaning that this type of steel is not suitable for functions that involve strain hardening. Different microalloying additions have been studied to further improve the microstructure of TWIP Steels [74].

If we compare resulting score values only for Al-based materials, porous Al has significantly different scores depending on the MCDM method, while two other observed alloys (M4—Al 6005 and M5—Al 6082) have almost the same final scores. Porous Al was ranked in third or fourth positions by the majority of the methods, except for PROMETHEE where it had the lowest (sixth) rank. The PROMETHEE II method considers the complete ranking by identifying the best criteria, followed by calculation of the preference indices in relation to the best criteria. It was expected that porous Al would get the lowest rank here, since its mechanical properties (yield and tensile strength) are far beyond steel and bulk aluminium (Table 2).

The weights for criteria comparison by using five different MCDM methods are set up to favour density (to provide light-weight components), ratio of yield to tensile strength (Y.S./T.S. ratio) and corrosion resistance (C.R.). It is obvious that changes of these weights would result in significantly different material rankings. The ratio of yield to tensile strength represents a significant material property that indicates a good safety margin against failure from deformation collapse. The Y.S./T.S. ratio is a measure of the ability for strain hardening and ductility, and higher values (over 0.5) indicate lower ability for strain hardening and lower material ductility [75]. Higher values of strength and the Y.S./T.S. ratio in advanced steels (M1, M2, M3 in Table 2) has been allowed in our study, aiming for materials that can withstand catastrophic events such as natural disasters (earthquakes, snowstorm or tornados) or functional catastrophic events (uch as collisions). The structural design of materials considers their functional component behaviour, so that for structures that will operate only in an elastic region (and can behave as fully elastic even at extreme load conditions, such as plain supported beams), the Y.S./T.S. ratio becomes an irrelevant property. In the case of structural components such as connections, link beams, or flanges, the Y.S./T.S. ratio is very relevant, because such components are expected to withstand stresses and strains in the strain-hardening range, and even more so in the necking range of loads.

Advanced steels and aluminium alloys have also been developed to provide better weldability and improved corrosion resistance (including weathering) [10,62,72,73,76,77]. Component weight was not a focus in the early days of steel improvements, until the use of aluminium introduced lightweighting in car body structures. However, even with low weight of Al-based structures, railway vehicles are still very heavy, thus fuel consumption is very high and development of porous Al-based materials has become a focus of research in recent years [4,25,26,78,79,80,81].

If we compare values of the Y.S./T.S. ratio for porous Al (Table 2), it can be noticed that it is comparable to these values for steel and slightly lower than for the other two Al alloys, meaning that is a good candidate for construction of beams and beam boxes in vehicles [82]. Sandwich panels have emerged as a material of choice for different applications, whereas higher values of the Y.S./T.S. ratio for porous Al than for uniform Al is experimentally validated [83]. It can be seen from our analysis that yield strength and tensile strength of porous Al are significantly lower that other material candidates, placing it in lower ranks than others, thus limiting its application as a structural material on its own. Moreover, its price is still very high, which is one of the significant barriers for its wider applications as well. 

**Figure 2:**
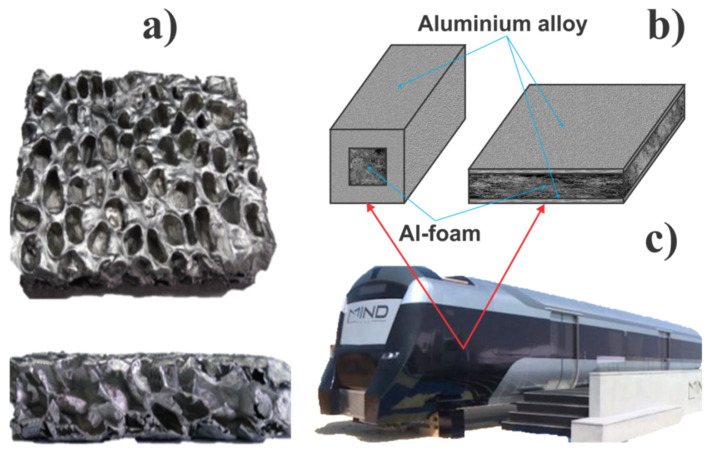
Porous aluminium (**a**) and its application in crash absorber boxes and sandwich panels (**b**) that has been used in railway vehicles (**c**).

Sandwich panels made of Al sheets with porous Al as the core material are good composite material for lightweight structural boxes in vehicles that can serve as shock and crash absorbers (Figure 2). They are also an excellent insulation material to provide fire protection, thermoregulation and sound proofing properties [84,85]. Energy absorption capabilities are excellent as well [86]. These types of composite structures can overcome the drawback of pure porous Al associated with low yield strength and tensile strength. Unlike porous Al structures, lightweight sandwich panels offer good mechanical strength and well balanced load-bearing structural properties, even though these are not primary properties demanded from sandwich panels. Al-based sandwich panels with a porous Al core have been studied as a lightweight material for electric vehicles also [82]. Beside its light weight, the capacity of the material to absorb energy is in high demand in vehicle design, since such material property significantly contributes to the prevention of crash at collisions [84]. High kinetic energy of the impact is transformed into strain energy via deformation mechanisms of the sandwich structure, thus allowing extensive amounts of high kinetic energy to be absorbed [86].

Figure 2 shows real elements of a railway wagon where aluminium foam has been used—train body panels, extendable door steps in the train, and crash absorber boxes. All of these elements provide several functions and with further research, these types of composite materials will become increasingly extensively used for other vehicle elements. Based on our analysis, it is clear that wider applications of porous Al would demand further improvements in its mechanical properties (both tensile and yield strength), as well as a decrease in its production cost. Material analysis based on MCDM methods clearly indicated areas of future improvements for each of these materials, but that depends also on their final application in specific components of the railway vehicles. In the case of metal foam for the core of sandwich panels, porous Al is an excellent candidate, which is in accordance with results from other material selection methods that can be found in the literature [87]. Further improvements of this type of material structure have been studied from different angles, focusing on specific issues such as interfacial debonding (along the contact between the porous core and uniform sheet) [88], wear resistance [89], and cost-efficiency and suitable production technology [80]. 

Our results showed that comparison of material ranking in different MCDM methods provide a better overview and starting point for suitable material selection, and such an approach also better addresses possible questions that can occur when using only one material selection method. Subjective assignment of weights in multi-criteria decision-making techniques also need further research and improvements towards objective and integrated weighting methods, [90], that will provide more reliable material selection recommendations [91,92,93]. On the other hand, a more complex approach would require more resources and skills from the decision-maker, even though it offers less bias potential [94]. Advancements in software development that will implement some new approach to automation [95,96] will bring about better MCDM methods, but the drawback is usually the high software costs. Hence, further research on material selection methods should consider different opposing requests, from rapid comparison, degree of expert opinion involvement and autonomous recommendations with less bias potential.

## 4. Conclusions

Multi-criteria Decision Making Methods (MCDM) were used for the selection of lightweight materials for railway vehicles. VIKOR, TOPSIS, PROMETTHEE and the Weighted aggregated sum product assessment method were applied on six different materials: advanced steel, aluminium alloys and porous aluminium structure. Dual-Phase 600 steel, Transformation-Induced Plasticity (TRIP) 700 steel, Twinning-Induced Plasticity (TWIP) steel, Aluminum, Al 6005-T6, Aluminum, Al 6082-T6, and porous Al structure with closed cells were analysed by considering their key properties: density, yield strength (Y.S.), tensile strength (T.S.), the Y.S./T.S. ratio, Youngs modulus (Y.M.), cost and corrosion resistance (C.R.).

Based on preferences toward corrosion resistance, modulus of elasticity and strength, aluminium alloys were the highest ranked materials. Lightweight aluminium alloys have proven their usefulness in railway vehicles, but advanced steels that we observed were also closely ranked, thus showing that they are also good candidates. However, porous aluminium was not ranked high in some MCDM methods, mainly due to its significantly lower strength, thus indicating that such material can be used in elements of railway vehicles that do not require load bearing.

## Figures and Tables

**Table 1:** Engineering material properties for MCDM investigation [60,61,62,63,64,65,66,67,68,69].

	Density	Yield Strength (Y.S.), MPa	Tensile Strength (T.S.), MPa	Y.S./T.S. Ratio	Youngs Modulus	Price US $/kg	Corrosion Resistance
	(kg/m^3^)				(GPa)		
Dual Phase,	8050	410	700	0.58	200	0.55	1
DP 600							
Transformation Induced Plasticity, TRIP 700	8050	520	800	0.5	200	0.55	1
Twinning Induced Plasticity, TWIP	8050	750	1000	0.75	200	1.5	1
Aluminium, Al 6005-T6	2700	240	260	0.9	69	1.9	3
Aluminium, Al 6082-T6	2700	250	310	0.88	70	1.9	3
Porous Structure (Al—Closed cell)	1000	20	30	0.66	12	46	3

**Table 2:** Chemical composition of DP 600, TRIP 700 and TWIP Steel [60,61,62,63,64,65,66,67,68,69].

	Max. C (%)	Max. Si (%)	Max. Mn (%)	Max. P (%)	Max. S (%)	Al (%)	Max. Cu (%)	Max. B (%)	Max. Ti + Nb (%)	Max. Cr + Mo (%)
DP 600	0.15	0.8	2.5	0.05	0.01	0.010	0.2	0.005	0.15	-
TRIP 700	0.24	2.0	2.2	0.05	0.01	0.015	0.2	0.005	0.2	0.6
TWIP-steel	0.60	0.60	25.0	0.03	0.005	2.50	0.20	-	0.20	2.50

**Table 3:** Chemical composition of Al 6005-T6 and Al 6082-T6 [17,22,23].

Component Element Properties	6005-T6 (%)	6082-T6 (%)
Aluminium, Al	97.5–99	95.2–98.3
Chromium, Cr	<=0.10	<=0.25
Copper, Cu	<=0.10	<=0.10
Iron, Fe	<=0.35	<=0.50
Magnesium, Mg	0.40–0.60	0.60–1.2
Manganese, Mn	<=0.10	0.40–1.0
Other, each	<=0.05	<=0.05
Other, total	<=0.15	<=0.15
Silicon, Si	0.60–0.90	0.70–1.3
Titanium, Ti	<=0.10	<=0.10
Zinc, Zn	<=0.10	<=0.20

**Table 4:** Properties of prospective material for light wagon railway engineering.

Properties of the Prospective Material	Symbol
Density, kg/m^3^	P_1_
Yield strength (Y.S.), MPa	P_2_
Tensile strength (T.S.), MPa	P_3_
Y.S./T.S. Ratio	P_4_
Youngs modulus (Y.M.), GPa	P_5_
Price, USD/kg	P_6_
Corrosion resistance (C.R.)	P_7_

**Table 5:** Engineering material alternatives.

Engineering Material Alternative	Symbol
Dual Phase, DP 600	M_1_
Transformation Induced Plasticity, TRIP 700	M_2_
Twinning Induced Plasticity, TWIP	M_3_
Aluminium, Al 6005-T6	M_4_
Aluminium, Al 6082-T6	M_5_
Porous Structure (Al—Closed cell)	M_6_

**Table 6:** Engineering materials with their properties along with best and worst values [60,61,62,63,64,65,66,67,68,69].

	Density (kg/ m^3^)	Yield Strength (Y.S.), MPa	Tensile Strength (T.S.), MPa	Y.S./T.S. Ratio	Youngs Modulus, GPa	Price, USD/kg	Corrosion Resistance
Dual Phase, DP 600	8050	410	700	0.58	200	0.55	1
Transformation Induced Plasticity, TRIP 700	8050	520	800	0.5	200	0.55	1
Twinning Induced Plasticity, TWIP	8050	750	1000	0.75	200	1.5	1
Aluminium, Al 6005-T6	2700	240	260	0.9	69	1.9	3
Aluminium, Al 6082-T6	2700	250	310	0.88	70	1.9	3
Porous Structure (Al—Closed cell)	1000	20	30	0.66	12	46	3
Best (Xi+)	1000	750	1000	0.9	200	0.55	3
Worst (Xi−)	8050	20	30	0.5	12	46	1

**Table 7:** Normalizing the evaluation matrix (decision matrix).

	P_1_	P_2_	P_3_	P_4_	P_5_	P_6_	P_7_
M_1_	0.200	0.047	0.015	0.160	0.000	0.000	0.3000
M_2_	0.200	0.032	0.010	0.200	0.000	0.000	0.3000
M_3_	0.200	0.000	0.000	0.075	0.000	0.001	0.3000
M_4_	0.048	0.070	0.038	0.000	0.070	0.001	0.0000
M_5_	0.048	0.068	0.036	0.010	0.069	0.001	0.0000
M_6_	0.000	0.100	0.050	0.120	0.100	0.050	0.000

**Table 8:** Calculating $S_{j}$, $R_{j}$ and $Q_{j}$—the utility measure, the regret measure and VIKOR index, respectively.

	*S*_i_	*R*_i_	*Q*_i_	Ranking
M_1_	0.7220	0.3000	0.9808	5
M_2_	0.7418	0.3000	1.0000	6
M_3_	0.5760	0.3000	0.8389	4
M_4_	0.2274	0.0699	0.0015	1
M_5_	0.2329	0.0691	0.0054	2
M_6_	0.4200	0.1200	0.2973	3

**Table 9:** Vector normalization of decision matrix.

	P_1_	P_2_	P_3_	P_4_	P_5_	P_6_	P_7_
M_1_	64,802,500	168,100	490,000	0.34	40,000	0.30	1
M_2_	64,802,500	270,400	640,000	0.25	40,000	0.30	1
M_3_	64,802,500	562,500	1,000,000	1	40,000	2	1
M_4_	7,290,000	57,600	67,600	1	4761	4	9
M_5_	7,290,000	62,500	96,100	1	4900	4	9
M_6_	1,000,000	400	900	0.44	144	2116	9

**Table 10:** Weighted normalized decision matrix with ideal best and ideal worst value.

	P_1_	P_2_	P_3_	P_4_	P_5_	P_6_	P_7_
M_1_	0.11	0.04	0.02	0.07	0.06	0.00	0.05
M_2_	0.11	0.05	0.03	0.06	0.06	0.00	0.05
M_3_	0.11	0.07	0.03	0.08	0.06	0.00	0.05
M_4_	0.04	0.02	0.01	0.10	0.02	0.00	0.16
M_5_	0.04	0.02	0.01	0.10	0.02	0.00	0.16
M_6_	0.01	0.00	0.00	0.07	0.00	0.05	0.16
V+	0.0138	0.0708	0.0330	0.1011	0.0555	0.0006	0.1643
V−	0.1111	0.0019	0.0010	0.0562	0.0033	0.0499	0.0548

V+ = Ideal Best, V− = Ideal Worst Value.

**Table 11:** Euclidean distance from ideal best (positive Si+) and worst (negative Si−) value and calculation of performance score (Pi).

	Si+	Si−	Pi	Rank
M_1_	0.1546	0.0841	0.3525	6
M_2_	0.1549	0.0896	0.3664	5
M_3_	0.1475	0.1078	0.4222	4
M_4_	0.0692	0.1500	0.6843	2
M_5_	0.0679	0.1496	0.6879	1
M_6_	0.1080	0.1476	0.5776	3

**Table 12:** Normalizing of the evaluation matrix (decision matrix).

	P_1_	P_2_	P_3_	P_4_	P_5_	P_6_	P_7_
M_1_	1.00	0.53	0.69	0.20	1.00	1.00	0.00
M_2_	1.00	0.68	0.79	0.00	1.00	1.00	0.00
M_3_	1.00	1.00	1.00	0.63	1.00	0.98	0.00
M_4_	0.24	0.30	0.24	1.00	0.30	0.97	1.00
M_5_	0.24	0.32	0.29	0.95	0.31	0.97	1.00
M_6_	0.00	−0.14	0.00	0.40	0.00	0.00	1.00

**Table 13:** Calculation of evaluative differences of *i*th alternative with respect to other alternatives.

	P_1_	P_2_	P_3_	P_4_	P_5_	P_6_	P_7_
D _M1-M2_	0.00	−0.15	−0.10	0.20	0.00	0.00	0.00
D _M1-M3_	0.00	−0.47	−0.31	−0.43	0.00	0.02	0.00
D _M1-M4_	0.76	0.23	0.45	−0.80	0.70	0.03	−1.00
D _M1-M5_	0.76	0.22	0.40	−0.75	0.69	0.03	−1.00
D _M1-M6_	1.00	0.67	0.69	−0.20	1.00	1.00	−1.00
D _M2-M1_	0.00	0.15	0.10	−0.20	0.00	0.00	0.00
D _M2-M3_	0.00	−0.32	−0.21	−0.63	0.00	0.02	0.00
D _M2-M4_	0.76	0.38	0.56	−1.00	0.70	0.03	−1.00
D _M2-M5_	0.76	0.37	0.51	−0.95	0.69	0.03	−1.00
D _M2-M6_	1.00	0.82	0.79	−0.40	1.00	1.00	−1.00
D _M3-M1_	0.00	0.47	0.31	0.43	0.00	−0.02	0.00
D _M3-M2_	0.00	0.32	0.21	0.63	0.00	−0.02	0.00
D _M3-M4_	0.76	0.70	0.76	−0.38	0.70	0.01	−1.00
D _M3-M5_	0.76	0.68	0.71	−0.33	0.69	0.01	−1.00
D _M3-M6_	1.00	1.14	1.00	0.23	1.00	0.98	−1.00
D _M4-M1_	−0.76	−0.23	−0.45	0.80	−0.70	−0.03	1.00
D _M4-M2_	−0.76	−0.38	−0.56	1.00	−0.70	−0.03	1.00
D _M4-M3_	−0.76	−0.70	−0.76	0.38	−0.70	−0.01	1.00
D _M4-M5_	0.00	−0.01	−0.05	0.05	−0.01	0.00	0.00
D _M4-M6_	0.24	0.44	0.24	0.60	0.30	0.97	0.00
D _M5-M1_	−0.76	−0.22	−0.40	0.75	−0.69	−0.03	1.00
D _M5-M2_	−0.76	−0.37	−0.51	0.95	−0.69	−0.03	1.00
D _M5-M3_	−0.76	−0.68	−0.71	0.33	−0.69	−0.01	1.00
D _M5-M4_	0.00	0.01	0.05	−0.05	0.01	0.00	0.00
D _M5-M6_	0.24	0.45	0.29	0.55	0.31	0.97	0.00
D _M6-M1_	−1.00	−0.67	−0.69	0.20	−1.00	−1.00	1.00
D _M6-M2_	−1.00	−0.82	−0.79	0.40	−1.00	−1.00	1.00
D _M6-M3_	−1.00	−1.14	−1.00	−0.23	−1.00	−0.98	1.00
D _M6-M4_	−0.24	−0.44	−0.24	−0.60	−0.30	−0.97	0.00
D _M6-M5_	−0.24	−0.45	−0.29	−0.55	−0.31	−0.97	0.00

**Table 14:** Calculation of preference functions *P_j_* (a, b).

	P_1_	P_2_	P_3_	P_4_	P_5_	P_6_	P_7_
D _M1-M2_	0.00	0.00	0.00	0.04	0.00	0.00	0.00
D _M1-M3_	0.00	0.00	0.00	0.00	0.00	0.00	0.00
D _M1-M4_	0.15	0.02	0.02	0.00	0.07	0.00	0.00
D _M1-M5_	0.15	0.02	0.02	0.00	0.07	0.00	0.00
D _M1-M6_	0.20	0.07	0.03	0.00	0.10	0.05	0.00
D _M2-M1_	0.00	0.02	0.01	0.00	0.00	0.00	0.00
D _M2-M3_	0.00	0.00	0.00	0.00	0.00	0.00	0.00
D _M2-M4_	0.15	0.04	0.03	0.00	0.07	0.00	0.00
D _M2-M5_	0.15	0.04	0.03	0.00	0.07	0.00	0.00
D _M2-M6_	0.20	0.08	0.04	0.00	0.10	0.05	0.00
D _M3-M1_	0.00	0.05	0.02	0.09	0.00	0.00	0.00
D _M3-M2_	0.00	0.03	0.01	0.13	0.00	0.00	0.00
D _M3-M4_	0.15	0.07	0.04	0.00	0.07	0.00	0.00
D _M3-M5_	0.15	0.07	0.04	0.00	0.07	0.00	0.00
D _M3-M6_	0.20	0.11	0.05	0.05	0.10	0.05	0.00
D _M4-M1_	0.00	0.00	0.00	0.16	0.00	0.00	0.30
D _M4-M2_	0.00	0.00	0.00	0.20	0.00	0.00	0.30
D _M4-M3_	0.00	0.00	0.00	0.08	0.00	0.00	0.30
D _M4-M5_	0.00	0.00	0.00	0.01	0.00	0.00	0.00
D _M4-M6_	0.05	0.04	0.01	0.12	0.03	0.05	0.00
D _M5-M1_	0.00	0.00	0.00	0.15	0.00	0.00	0.30
D _M5-M2_	0.00	0.00	0.00	0.19	0.00	0.00	0.30
D _M5-M3_	0.00	0.00	0.00	0.07	0.00	0.00	0.30
D _M5-M4_	0.00	0.00	0.00	0.00	0.00	0.00	0.00
D _M5-M6_	0.05	0.05	0.01	0.11	0.03	0.05	0.00
D _M6-M1_	0.00	0.00	0.00	0.04	0.00	0.00	0.30
D _M6-M2_	0.00	0.00	0.00	0.08	0.00	0.00	0.30
D _M6-M3_	0.00	0.00	0.00	0.00	0.00	0.00	0.30
D _M6-M4_	0.00	0.00	0.00	0.00	0.00	0.00	0.00
D _M6-M5_	0.00	0.00	0.00	0.00	0.00	0.00	0.00

**Table 15:** Aggregated preference for different materials for the calculation of the leaving flow.

Aggregated Preference Functions	Dual Phase, DP 600	Transformation-Induced Plasticity, TRIP 700	Twinning-Induced Plasticity, TWIP	Aluminium, Al 6005-T6	Aluminium, Al 6082-T6	Porous Structure (Al—Closed Cell)	Aggregated Value	Leaving Flow+
Dual Phase, DP 600	0.000	0.040	0.001	0.269	0.264	0.452	1.0262	0.2052
Transformation-Induced Plasticity, TRIP 700	0.020	0	0.001	0.289	0.285	0.472	1.0671	0.2134
Twinning-Induced Plasticity, TWIP	0.147	0.167	0	0.330	0.325	0.558	1.5270	0.3054
Aluminium, Al 6005-T6	0.460	0.500	0.375	0	0.010	0.303	1.6480	0.3296
Aluminium, Al 6082-T6	0.450	0.490	0.365	0.004	0	0.297	1.6069	0.3214
Porous Structure (Al—Closed cell)	0.340	0.380	0.300	0.000	0.000	0	1.0200	0.2040
Aggregated value	1.4173	1.5768	1.0421	0.8924	0.8845	2.0822		
Entering Flow−	0.2835	0.3154	0.2084	0.1785	0.1769	0.4164		

**Table 16:** Ranking of material according to leaving, entering and outranking flow.

	Leaving Flow+	Entering Flow−	Net Flow	Rank
Dual Phase, DP 600	0.2052	1.4173	−1.2120	4
Transformation-Induced Plasticity, TRIP 700	0.2134	1.5768	−1.3634	5
Twinning-Induced Plasticity, TWIP	0.3054	1.0421	−0.7367	3
Aluminium, Al 6005-T6	0.3296	0.8924	−0.5628	1
Aluminium, Al 6082-T6	0.3214	0.8845	−0.5631	2
Porous Structure (Al—Closed cell)	0.2040	2.0822	−1.8782	6

**Table 17:** Normalized decision matrix values.

	P1	P2	P3	P4	P5	P6	P7
M1	0.124224	0.547	0.7	0.644	1	1	0.333
M2	0.124224	0.693	0.8	0.56	1	1	0.333
M3	0.124224	1	1	0.83	1	0.37	0.333
M4	0.370224	0.32	0.26	1	0.34	0.289	1
M5	0.37037	0.33	0.31	0.977	0.35	0.289	1
M6	1	0.027	0.03	0.733	0.06	0.011	1

**Table 18:** Values obtained by weighted normalized decision matrix using the sum method.

	P1	P2	P3	P4	P5	P6	P7
M1	0.024845	0.0547	0.035	0.01289	0.1	0.05	0.1
M2	0.024845	0.0693	0.04	0.0111	0.1	0.05	0.1
M3	0.024845	0.1	0.05	0.1667	0.1	0.01833	0.1
M4	0.074074	0.032	0.013	0.2	0.0345	0.01447	0.3
M5	0.074074	0.0333	0.0155	0.1956	0.035	0.01447	0.3
M6	0.2	0.0027	0.0015	0.1467	0.006	0.00059	0.3

**Table 19:** Values obtained by weighted normalized decision matrix using the product method.

	P1	P2	P3	P4	P5	P6	P7
M1	0.6589	0.94139	0.98232	0.91588	1	1	0.71922
M2	0.6589	0.96404	0.98890	0.88909	1	1	0.71922
M3	0.6589	1	1	0.96419	1	0.95107	0.71922
M4	0.8198	0.89231	0.93486	1	0.89905	0.93989	1
M5	0.8198	0.89595	0.94312	0.99552	0.90034	0.93989	1
M6	1	0.69598	0.83918	0.93985	0.75477	0.80146	1

**Table 20:** Performance score obtained by weighted normalized decision matrix using the sum method and product method.

	Performance Score Using Product Method	Performance Score Using Sum Method	WASPAS Performance Score	WASPAS Ranking
M1	0.40139	0.493400	0.447396	6
M2	0.40169	0.495289	0.448493	5
M3	0.43459	0.559844	0.497218	3
M4	0.57790	0.668047	0.622974	2
M5	0.58360	0.667936	0.625770	1
M6	0.33205	0.657431	0.494743	4

**Table 21:** Ranking of material using MCDM methods.

	TOPSIS		WASPAS		VIKOR		PROMETHEE	
	Score	Rank	Score	Rank	Score	Rank	Score	Rank
M1	0.3525	6	0.447396	6	0.9808	5	−1.2120	4
M2	0.3664	5	0.448493	5	1.0000	6	−1.3634	5
M3	0.4222	4	0.497218	3	0.8389	4	−0.7367	3
M4	0.6843	2	0.622974	2	0.0015	1	−0.5628	1
M5	0.6879	1	0.625770	1	0.0054	2	−0.5631	2
M6	0.5776	3	0.494743	4	0.2973	3	−1.8782	6
